# The translational power of Alzheimer’s-based organoid models in personalized medicine: an integrated biological and digital approach embodying patient clinical history

**DOI:** 10.3389/fncel.2025.1553642

**Published:** 2025-05-15

**Authors:** Cristina Dolciotti, Marco Righi, Eleonora Grecu, Marcello Trucas, Cristina Maxia, Daniela Murtas, Andrea Diana

**Affiliations:** ^1^Department of Translational Research and New Technologies in Medicine and Surgery, University of Pisa, Pisa, Italy; ^2^Clinical Physiology Institute, The Italian National Research Council (CNR), Massa, Italy; ^3^Department of Biomedical Sciences, University of Cagliari, Cagliari, Italy

**Keywords:** Alzheimer’s disease, neurodegeneration, personalized medicine, brain organoids, early diagnosis biomarker, neuroinflammation, digital twins

## Abstract

Alzheimer’s disease (AD) is a complex neurodegenerative condition characterized by a multifaceted interplay of genetic, environmental, and pathological factors. Traditional diagnostic and research methods, including neuropsychological assessments, imaging, and cerebrospinal fluid (CSF) biomarkers, have advanced our understanding but remain limited by late-stage detection and challenges in modeling disease progression. The emergence of three-dimensional (3D) brain organoids (BOs) offers a transformative platform for bridging these gaps. BOs derived from patient-specific induced pluripotent stem cells (iPSCs) mimic the structural and functional complexities of the human brain. This advancement offers an alternative or complementary approach for studying AD pathology, including β-amyloid and tau protein aggregation, neuroinflammation, and aging processes. By integrating biological complexity with cutting-edge technological tools such as organ-on-a-chip systems, microelectrode arrays, and artificial intelligence-driven digital twins (DTs), it is hoped that BOs will facilitate real-time modeling of AD progression and response to interventions. These models capture central nervous system biomarkers and establish correlations with peripheral markers, fostering a holistic understanding of disease mechanisms. Furthermore, BOs provide a scalable and ethically sound alternative to animal models, advancing drug discovery and personalized therapeutic strategies. The convergence of BOs and DTs potentially represents a significant shift in AD research, enhancing predictive and preventive capacities through precise *in vitro* simulations of individual disease trajectories. This approach underscores the potential for personalized medicine, reducing the reliance on invasive diagnostics while promoting early intervention. As research progresses, integrating sporadic and familial AD models within this framework promises to refine our understanding of disease heterogeneity and drive innovations in treatment and care.

## 1 Introduction

The diagnosis of Alzheimer’s disease (AD) integrates medical history, neuropsychological assessments, imaging, and cerebrospinal fluid (CSF) analysis. A detailed clinical history examines cognitive and behavioral symptoms, progression, and risk factors (age, family history, and any comorbidities). Differential diagnoses help out in predicting pseudodementia, frontotemporal dementia, Lewy body dementia, and vascular dementia (Thangwaritorn et al., 2024). Neuropsychological assessments, such as the Mini-Mental State Examination (MMSE) (Arevalo-Rodriguez et al., 2021) and Montreal Cognitive Assessment (MoCA) (Ciesielska et al., 2016), provide a global cognitive screening, whereas specific tests assess memory, executive functions, and language. Clinical scales including Activities of Daily Living (ADL), Instrumental Activity of Daily Living (IADL) (Teng et al., 2023), and the Clinical Dementia Rating (CDR) (Cummings et al., 2023), evaluate functional autonomy and dementia severity. The CDR stages range from normal aging (CDR 0) to severe dementia (CDR 3), aiding in distinguishing Mild Cognitive Impairment (MCI) (CDR 0.5) from AD (Gkenios et al., 2022).

Imaging plays a pivotal role in diagnosis. Magnetic Resonance Imaging (MRI) allows to detect AD-related hippocampal and cortical atrophy, particularly in the temporo-parietal regions (Chandra et al., 2019). Positron emission tomography (PET) with fluorodeoxyglucose (FDG) reveals hypometabolism in medial temporal and parietal regions, while amyloid PET highlights β-amyloid deposition, a well-known hallmark of AD (Chételat et al., 2020). All of them are extremely informative in early or atypical disease presentations.

CSF biomarkers such as reduced β-amyloid 42 (Aβ42), increased total tau (T-Tau), and phosphorylated tau (P-Tau), are crucial for biological AD confirmation. The Aβ42/Aβ40 ratio enhances diagnostic precision, while additional biomarkers address neuroinflammation and oxidative stress (Olsson et al., 2016; Ossenkoppele et al., 2022; Dubois et al., 2023: Pascoal et al., 2024).

A potential AD diagnosis requires clinical symptoms and at least one positive biomarker, while a possible diagnosis applies to typical symptoms and inconclusive biomarkers. Definitive diagnosis is achieved post-mortem via neuropathological analysis. AD diagnosis remains challenging due to its overlap with mixed or vascular dementias, which necessitates a nuanced diagnostic approach (Zekry et al., 2002).

AD also embraces a spectrum of preclinical stages. Subjective Cognitive Impairment (SCI) and Mild Cognitive Impairment MCI represent prodromal conditions. SCI involves perceived cognitive deficits with neuroimaging evidence (Stewart, 2012; Wang et al., 2020), in contrast to MCI that denotes measurable cognitive decline with preserved function. MCI is a significant risk factor for AD, though not all cases progress to dementia (Jongsiriyanyong and Limpawattana, 2018).

Integrating clinical, neuropsychological, imaging, and biochemical biomarkers enhances diagnostic accuracy and informs targeted therapies. However, AD diagnosis often occurs late relative to underlying neurobiological changes, emphasizing the need for early identification and intervention.

Finally, we are now entering a pivotal era in which the evaluation of biomarkers in presymptomatic individuals will offer the biological foundation necessary to transition conventional plasma biomarkers into digital biomarkers (Cash et al., 2025; Aghdam et al., 2025; Jiao et al., 2025).

## 2 Exploring new frontiers in Alzheimer’s disease diagnosis: beyond the brain

Recommendation frameworks for diagnosing AD have been recently updated by the National Institute of Aging and the Alzheimer’s Association (NIA-AA) (Jack et al., 2024), based on the latest advancements that refine the previous document (Jack et al., 2018). On this matter, AD definition can be declined as a biological process that begins with the emergence of Alzheimer’s disease neuropathologic change (ADNPC) while individuals are still asymptomatic. As the neuropathologic burden progresses, clinical symptoms eventually appear and worsen. Early-changing biomarkers, such as amyloid PET-highlighted changes, CSF biomarkers, and p-Tau 217 (core 1 biomarker), indicate the presence of ADNPC. An abnormal result from a core 1 biomarker is sufficient for diagnosing AD and guiding clinical decisions. Later-changing core 2 biomarkers, including biofluids and tau PET, provide prognostic data that assist with confirming AD’s role in symptoms. The main goal is to establish objective criteria for diagnosing and staging AD thanks to the new advancements in biomarkers, to bridge the gap between research and clinical care settings. Currently, while the use of biomarkers enhances the *in vivo* diagnosis of AD, the approach has shifted from a syndromic model to a biological one, based on the [AT (N)] classification developed by the NIA-AA, that categorizes patients based on the presence of amyloid (A), tau protein (T), and neurodegeneration (N) (Jack et al., 2018). The ATN system is designed to be flexible, allowing for the addition of central and peripheral biomarkers as they become available. This is why some researchers advocate for including further molecular biomarkers, particularly those related to inflammation, in both the central nervous system (Xc) and the periphery (Xp) (Huang et al., 2022). This shifting paradigm aims to identify novel or unconventional diagnostic and prognostic biomarkers (Klyucherev et al., 2022) beyond the brain, especially in blood (Hampel et al., 2023) and CSF, or other biological fluids (Carmona-Iragui et al., 2024) to strengthen predictive and preventive strategies for AD (Khoury and Ghossoub, 2019; Baldini et al., 2022; Teunissen et al., 2022). For instance, cholesterol and its derivatives have emerged as significant biomarkers, linked to an increased risk of dementia in initially healthy older adults (Hussain et al., 2024). Moreover, microRNAs (miRNAs), crucial regulators of gene expression, have garnered attention for their potential clinical significance (Wei et al., 2020; Luo et al., 2022). Distinct miRNA profiles in the blood are valuable candidates for drawing correlations with various stages of AD, positioning them as useful targets for future interventions (Zhou et al., 2020; Bhatnagar et al., 2023). In the context of circulating and measurable chemical signals, recruiting exosomes and extracellular vesicles (EVs) containing miRNAs in the bloodstream has reinforced their potential as biomarkers for AD (Soares Martins et al., 2022; Abidin et al., 2023). By exploring biomarkers outside the traditional realms, researchers are paving the way for more comprehensive and early detection methods for AD, its clear-cut staging, and its discrimination against non-AD type dementias, offering hope for more effective treatment and prevention tools. Within this context, it is widely acknowledged that neuroinflammation plays a pivotal role in AD pathology. This pathway involves astrocytic and microglial activation, cytokine release, and alterations in the clearance of misfolded protein aggregates, all of which influence the onset and progression of AD. For instance, reactive astrogliosis (RA) exhibits a dual impact. While contributing to neurotoxicity and inflammation, it protects against neurotoxic agents and supports blood–brain barrier (BBB) repair (Singh, 2022). Moreover, systemic inflammasome activation (Rui et al., 2021) triggers a self-sustaining loop that leads the healthy brain to the Alzheimer’s manifestation through a cognitive impairment and finally to the Alzheimer’s manifestation, as recently confirmed by the role of the aberrant expression of Nucleotide-binding oligomerization domain (NOD), Leucine rich Repeat and Pyrin domain containing (NLRP) inflammasome activated caspase-1 (Beder et al., 2024). This statement intensifies the urgency of preclinical models, filling the gap between inconsistent preclinical AD animal models and the clinical appearance of the disease in humans.

## 3 The need for innovative experimental models: toward the modern age of 3D brain organoids

Despite their contributions to understanding AD mechanisms, both traditional animal models and transgenic ones fail to replicate the human brain’s intricate tissue structure, function, cellular diversity and pathology hallmarks as well as two-dimensional (2D) cell cultures merely contribute to the dissection of the involved molecular pathways (Hossain et al., 2024). In the last few years, ethical concerns and technical challenges have limited the study of the interactions between the human brain and peripheral organs; it has prompted the exploration of new experimental tools that could eventually reflect the brain exposure to several molecules, especially during the deterioration of BBB responsible for conveying harmful components to the already damaged brain (Chen et al., 2021).

The emergence of three-dimensional (3D) brain organoids (BOs), lab-grown structures developed through tissue engineering, have disclosed a revolutionary alternative to traditional AD models (Gonzalez et al., 2018; Kim et al., 2024). These mini encephalic organs mimic the *in vivo* physiology of the human brain, providing a more accurate representation of its structure and function (Hong et al., 2022; Jeong et al., 2023). Starting from blood or fibroblast samples and passing through the intermediate transformation into induced pluripotent stem cells (iPSCs) (Raja et al., 2016; Choe et al., 2024), various protocols for developing 3D BOs have flourished, differing in chemical composition, microenvironmental settings, and technological materials (Kwak et al., 2024). Indeed, whole-brain organoids and more specifically BOs of different regions can be built (e.g., dorsal forebrain or cerebral organoids (COs), respectively) (Del Dosso et al., 2020). However, over the past decade, more than 2,255 papers on “human brain organoids” have been published, underscoring the urgent need for a more unifying experimental methodology. These *in vitro* organs could really be leveraged for the advancement of basic and applied neuroscience only when comprehensive guidance and assertive advice on the design, execution, and sharing of experiments significantly will improve the reproducibility and utility of these models (Pașca et al., 2024). Recent findings have highlighted the importance of BOs in studying the fundamental mechanisms underlying AD. Notably, when these organoids are derived from AD patients, they retain biomarkers associated with the disease (Yan et al., 2018; Alić et al., 2021; Chen et al., 2021), even after undergoing a process of embryonic resetting through the use of iPSCs as intermediate cellular elements.

## 4 Patient-derived advanced BOs: combining biological and technological tools

The BOs exhibit certain features of human brain structure and AD-like pathology, serving as a tool to explore the connection between Alzheimer’s pathology and neural cell dysfunction that leads to cognitive decline. The morphological and biochemical analysis of BOs aligned with individual clinical histories creates a promising platform for modeling the patient-specific disease staging. Such experimental models offer the disease *continuum* from the incoming neurological deficits to AD diagnosis, which is still missing in the *in vitro* models despite the lack of vascularization and incomplete maturation. Meanwhile, at some point in the experimental study, BOs could express different recognized biomarkers, signaling the onset of the neurodegenerative process even when the clinical evidence is absent in the corresponding donor subjects and, thus, preventing *in vivo* invasive and expensive investigations. Furthermore, this innovative approach during the clinical follow-up will allow researchers to generate the clinically corresponding mini-brains to track clinical evolution. In contrast to clinical evaluations, the study of BOs and any biological fluids potentially analyzed for peripheral biomarkers can be carried out both prospectively and retrospectively, due to the possibility of storing them in certified biobanks.

**Figure 1:**
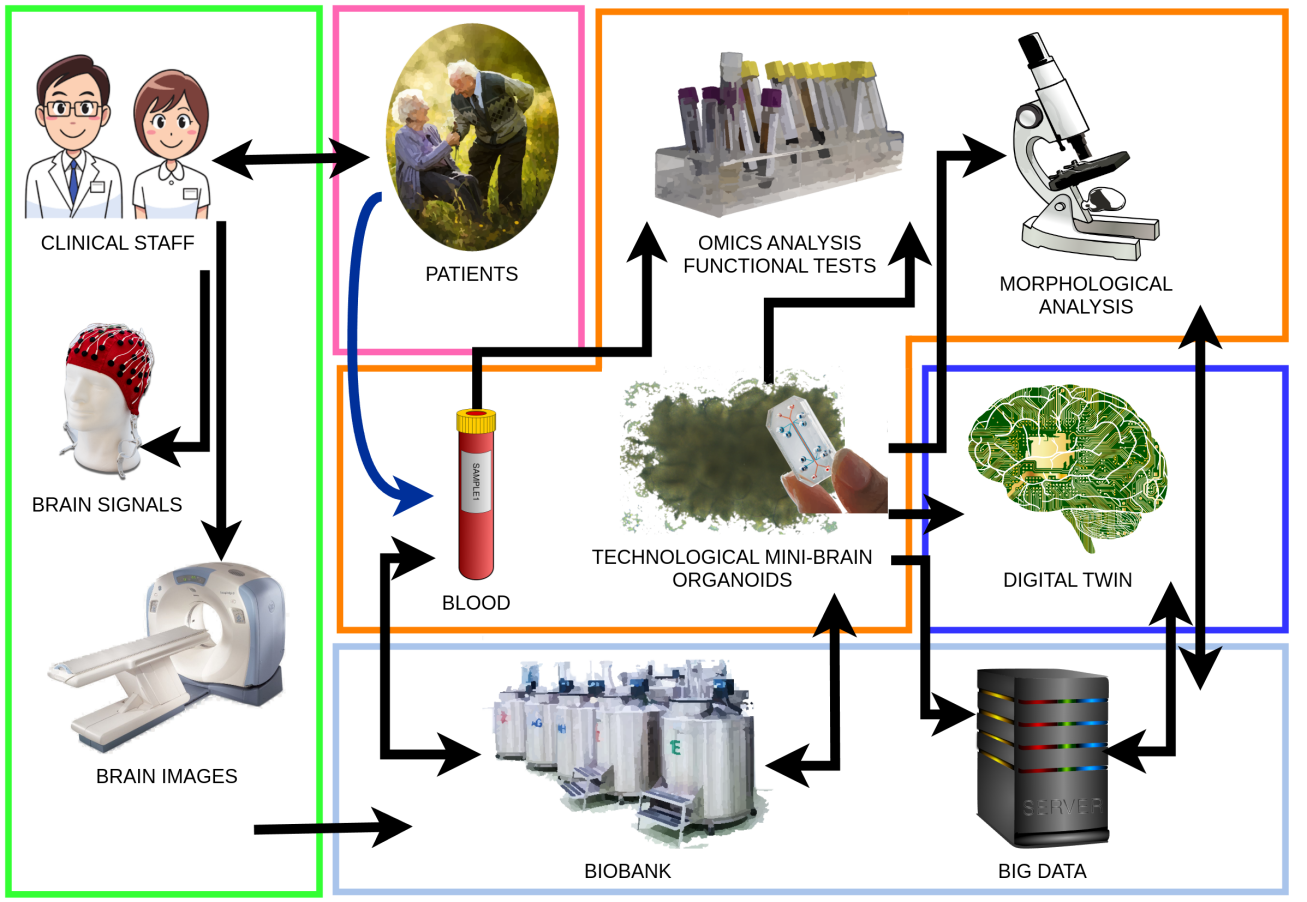
Brain organoids work-flow from patients to brain organoids and digital twins. Clinical staff: patient evaluation and classification through standard clinical tools and brain imaging. Blood: blood sampling, peripheral blood mononuclear cell extraction, and obtaining pluripotent stem cells to generate brain organoids. Technological mini-brain organoids: development and application of biotechnologies to achieve the structural complexity and cellular diversity of brain organoids. Analysis: diachronic execution of morphological analyses, omics analyses, and functional tests aimed at identifying AD biomarkers in brain organoids and blood of the organoid donor patients. Digital twin: creation of a mathematical model of the brain organoid that parallels its growth and degeneration. Data collection: collection of biological samples in a dedicated biobank and synthetic data, big data, in an open-access platform.

A comprehensive understanding of AD through BOs hinges on merging cutting-edge biological and technological advancements (Boylin et al., 2024). We visually represent this in our proposed model of investigation (Figure 1). In the context of the biological field, these advancements aim to fulfill several critical objectives:

1. *Recreating architectural complexity*: BOs will replicate the cellular heterogeneity of the central nervous system (CNS), encompassing neuronal and glial phenotypes, and incorporating vascular components like endothelial cells to preserve BBB integrity.
2. *Identifying CNS biomarkers*: BOs will undergo molecular and morphological analyses to identify validated or potential AD biomarkers, previously detected in corresponding *in vivo* anatomical structures, including toxic protein aggregates such as β-amyloid and tau proteins, which are crucial for comparing AD hallmarks *in vivo*.
3. *Correlating central and peripheral biomarkers*: despite the lack of direct connections to other organs, BOs will reveal temporal patterns within the aging organoids and potential correlations between identified central biomarkers and peripheral ones found in blood samples of the same patients.
4. *Analyzing immune responses*: recruitment and pathological transformation of resident immune cells (astrocytes and microglia) will be qualitatively and quantitatively assessed to understand their detrimental pro-inflammatory effects.
5. *Modeling aging processes*: induction of cellular senescence in BOs will simulate gene expression changes and epigenetic modifications during aging, a significant risk factor for neurodegenerative diseases.

To ensure reproducibility and increase predictive capabilities, the following technological tools will be employed:

1. *3D microelectrode arrays, and high*-*resolution electrophysiology devices*: they can record rhythmic activity with high spatio-temporal resolution and collectively synchronized electric signals.
2. *Organ-on-a-chip devices*: these devices will simulate physiological environments to optimize BO growth and homeostasis maintenance or alteration (see BBB defects).
3. *Biocomputing simulations*: utilizing computational models they can predict organoid behavior and response under varying conditions.
4. *Optimized algorithms*: the development of algorithms tailored for *in silico*-BOs interaction modeling will deepen the understanding of AD progression.
5. Creating Digital Twins (DTs) using artificial intelligence (AI).

The term “digital twin” (DT) was introduced by Grieves (2019) to describe a digital entity capable of replicating certain properties of a physical object. Unlike traditional computer simulations, which rely on static mathematical models, DTs are virtual replicas of physical entities continuously updated with real-time data. This capability enables real-time monitoring, analysis, and intervention, making DTs more dynamic and interactive than conventional simulations. The development of these models relied heavily on imaging technologies and computational frameworks to mimic the structure and basic functions of organs. These early-stage DTs served as proof-of-concept demonstrations, showing how virtual models could enhance decision-making in medical practice.

AI-driven DTs have been applied in various medical and healthcare fields, including stroke progression (Allen et al., 2021), tissue culture (Möller and Pörtner, 2021), neurosurgery (Chumnanvej et al., 2024), and personalized dementia care (Wickramasinghe et al., 2022). Initially focused on simulating specific organs to address clinical challenges, DT technology has evolved to support personalized medicine by predicting patient-specific treatment responses. Early applications involved creating digital replicas of organs, such as the heart and lungs to enhance surgical procedures and drug effectiveness before real-world implementation (Vallée, 2023; El-Warrak and de Farias, 2024).

A major leap in DT technology came with the incorporation of molecular and genetic data, particularly transcriptomics which enables the analysis of gene expression patterns underpinning cellular and organ functions (Hansen et al., 2024). By integrating such data, DTs moved beyond anatomical simulations to model the intricate biological processes within organs clarifying disease mechanisms. Moreover, the development of validation models is enhanced by advanced predictive features. For instance, heart digital twins can predict arrhythmias, enabling personalized interventions (Thangaraj et al., 2024). Liver models provide valuable insights into disease progression, which helps optimize treatments and improve patient outcomes (Subramanian, 2020). Similarly, brain digital twins simulate neurological conditions, assisting in the planning of accurate and safe surgeries (Fekonja et al., 2024). Finally, the convergence of organoid technology and DT frameworks is one of the most exciting advancements in this field, pushing the boundaries for studying disease pathology, drug responses, and developmental biology (Heydari et al., 2021).

When combined with DT technology, organoids might offer an unparalleled platform for modeling complex biological systems and could provide unique insights into how certain genetic mutations or environmental factors influence developmental pathways and gain functions.

Nevertheless, DT technology and its application to organoids face several challenges. One major limitation is the computational complexity involved in creating accurate and scalable models. Simulating the intricate interplay of genetic, molecular, and physiological processes within an organoid requires significant computational power and advanced algorithms making the integration of diverse data types—such as transcriptomics, proteomics, and metabolomics—into a unified DT a concrete technical hurdle. However, the high computational demands of these advanced models can be effectively mitigated through the power of quantum computing, particularly with technologies such as the Majorana I processor, which offers unprecedented capabilities in handling such complexity (Nanda et al., 2024).

Another challenge is the need for standardized protocols and frameworks to ensure the reproducibility and reliability of DT models. Establishing regulatory guidelines and ethical standards will be critical as these technologies move closer to clinical application.

By improving and leveraging AI-driven analytics, researchers can automate the integration and interpretation of complex datasets, thereby enhancing the scalability and accuracy of DTs. Additionally, the development of more sophisticated organoid cultures—such as vascularized or multi-organ systems—will further expand the capabilities of DTs in simulating human biology.

## 5 Discussion

Preclinical studies currently rely on *in vivo* animal models, including transgenic and aged specimens, with 8,624,692 animals sacrificed for scientific purposes in the EU and Norway in 2020 (Commission Staff Working Document, 2023). BO-based research aims to reduce the use of these models, facilitating faster and easier scaling of therapeutic interventions for AD. We propose an alternative methodology using a virtual environment to test drug candidates before clinical trials, which lowers costs, minimizes ethical concerns of animal testing, and allows for individualized treatment strategies. Individual-derived BOs serve as valuable *ex vivo* targets for monitoring pharmacological interventions during disease progression, offering insights that may lead to personalized therapies.

In clinical applications related to AD and MCI, bridging the gap between genetic predispositions and sporadic factors remains essential. Understanding these complexities is crucial for developing effective interventions aimed at preserving mental acuity and mitigating AD progression. Modeling AD with BOs has primarily focused on familial AD cases. However, there is a growing need to include sporadic cases to enhance representativeness and accurately recapitulate the pathology (Selkoe and Hardy, 2016). Recently, some authors have outlined the minimal and ideal recommended standards for the quantitative analysis of organoids, focusing on ensuring rigor and reproducibility in human BOs research (Sandoval et al., 2024). While we refrain from replicating issues already exposed in a recent exhaustive paper (Cerneckis et al., 2023), we wish to warn the scientific community about some caveats and potential troubleshooting issues when translating findings to AD-affected patients.

One notable contradiction involves the aging process, a fundamental and widely recognized risk factor for neurodegenerative diseases. COs from AD patients are formed through the differentiation of embryonic stem cells (ESCs) or iPSCs, which, in turn, are derived from AD fibroblasts or blood cells via genetic reprogramming (Kunitomi et al., 2022). However, due to reasons that are both time-consuming and costly, studies based on organoid technology for AD research often rely on a limited number of iPSC cell lines, in stark contrast to the significantly larger number of patients enrolled in clinical studies. This raises the critical question about the robustness of the observed differences genuinely associated with AD-related pathological symptoms. An incomplete but promising solution would be to invest in research programs that use iPSCs derived from patients with sporadic AD. This approach could help create a more solid and reliable experimental design, aiming to minimize biochemical and morphological differences between various batches of cells.

For example, in a recent study by Lee et al. (2022), while certain features such as Aβ deposits aligned with histopathological findings, the expression levels of AD-related genes Microtubule-Associated Protein Tau (MAPT) and Amyloid Precursor Protein (APP) were similar when comparing iPSCs derived from normal and familial AD patients. Moreover, neuronal excitation tested by electrophysiological recording was downregulated in the AD group, in stark contrast to previous reports (Lin et al., 2018; Ghatak et al., 2019). This suggests that a 3-month culture period may be insufficient to fully capture AD’s histopathological hallmarks, but longer *in vitro* timelines are strongly dependent on sophisticated and reliable microfluidic systems to ensure the same vitality degree to the overall aged BOs. Research on the aging process has already been conducted, as the *in vitro* timeline does not match the *in vivo* aging pathway (Gordon et al., 2021; Hossain et al., 2024; Park et al., 2025). In addition, even though there is a close overlapping between the transcriptome/epigenome of BOs and human primary fetal tissues (Amiri et al., 2018), more detailed studies are needed for a faithful comparison between BOs and the aging brain, both in normal and pathological conditions. Thus, while human brain organoids as research tools provide advantages for studying AD, they also present numerous controversies and weaknesses that must be addressed (Sainz et al., 2025). Finally, the evolution of DT technology from simple anatomical models to complex simulations of BOs acts as a transformative shift in healthcare and biomedical research. The standpoint of this review is that DTs of BOs could be part of a fruitful strategy designed to a better comprehension and interpretation of biological phenomena underpinning AD appearance and progression. By integrating molecular data and leveraging organoid platforms, DTs would offer unprecedented opportunities for studying AD and possible *in silico* therapeutic personalized intervention prior, in alternative, or in parallel with human clinical trials.
